# University students describe how they adopt AI for writing and research in a general education course

**DOI:** 10.1038/s41598-025-92937-2

**Published:** 2025-03-14

**Authors:** Rebecca W. Black, Bill Tomlinson

**Affiliations:** Department of Informatics, University of California, Irvine, Irvine, CA USA

**Keywords:** Artificial intelligence, ChatGPT, Education, Undergraduate education, Computer science, Information technology, Software

## Abstract

University students have begun to use Artificial Intelligence (AI) in many different ways in their undergraduate education, some beneficial to their learning, and some simply expedient to completing assignments with as little work as possible. This exploratory qualitative study examines how undergraduate students used AI in a large General Education course on sustainability and technology at a research university in the United States in 2023. Thirty-nine students documented their use of AI in their final course project, which involved analyzing conceptual networks connecting core sustainability concepts. Through iterative qualitative coding, we identified key patterns in students’ AI use, including higher-order writing tasks (understanding complex topics, finding evidence), lower-order writing tasks (revising, editing, proofreading), and other learning activities (efficiency enhancement, independent research). Students primarily used AI to improve communication of their original ideas, though some leveraged it for more complex tasks like finding evidence and developing arguments. Many students expressed skepticism about AI-generated content and emphasized maintaining their intellectual independence. While some viewed AI as vital for improving their work, others explicitly distinguished between AI-assisted editing and their original thinking. This analysis provides insight into how students navigate AI use when it is explicitly permitted in coursework, with implications for effectively integrating AI into higher education to support student learning.

## Introduction

Artificial Intelligence (AI) (Text throughout this article was written in collaboration with the ChatGPT (4o, https://chatgpt.com/) and Claude AI (3.5 Sonnet, https://claude.ai/) systems. The core argument, all references to external sources, and all data analysis, were conducted by the authors without AI support. All AI-produced content has been thoroughly revised and edited by the authors such that it reflects their own thinking on the topic of the article. We wrote this article in line with current guidelines from Springer^1^ and in line with best practices for writing with AI^2,3^.) has permeated many spheres of human life, shaping our interaction with technology and fundamentally transforming many traditionally generative practices. In higher education, the impact has been particularly profound, as students and educators increasingly use AI tools to support learning processes, aid in assignment completion, and enhance productivity^4^. Recent research shows that university students are adopting AI for various academic tasks, from writing assistance^5^ to peer-like feedback^6^ to interactive learning support^7^.

Understanding how students choose to use AI tools when given explicit permission is crucial for several reasons. First, as AI becomes more integrated into academic work, educators need evidence-based insights about effective integration strategies. Recent studies have shown that university students value autonomy and interactivity in their AI interactions^5^ and may prefer AI-generated feedback for certain tasks^8^. Second, there are important questions about how students maintain intellectual independence while leveraging AI support. Finally, as institutions develop policies around AI use, understanding actual student practices and perspectives becomes essential for creating meaningful guidelines.

Our study contributes to this research domain by examining student AI use during a pivotal moment: June 2023, just months after ChatGPT’s release. Through analysis of documented AI use in a large General Education course, we identify patterns in both technical and conceptual applications of AI tools. Our findings reveal some of the complexities of AI integration, where students often distinguish between using AI for mechanical tasks versus deeper conceptual work. While focused on a single course, this snapshot of early LLM adoption in higher education helps document and analyze how students used these forms of AI when explicitly permitted, with implications for developing effective educational practices and policies.

## Purpose and research questions

This study examines how undergraduate students use AI when its use is explicitly permitted in coursework. Our research questions were:

- How do students choose to use AI tools when given permission to do so in their academic work?
- What patterns emerge in students’ documented AI use across different aspects of their coursework?
- How do students perceive and evaluate their use of AI in completing academic tasks?

These questions aim to understand both the practical applications and student perspectives during early AI adoption in higher education.

### Background

The intersection of AI and education has been a major focus of research in recent years, as AI-driven tools continue to reshape educational practices. AI’s integration into education has been explored across a variety of domains, from its role in personalizing learning experiences^9^ to its applications in automating grading^10^ and enhancing student engagement through interactive learning environments^7^.

The introduction of ChatGPT^11^ in late 2022 marked a turning point in AI-driven education, significantly accelerating interest in its potential as a personalized learning tool. Unlike traditional AI-based tutoring or assessment systems that operate within predefined frameworks, ChatGPT’s ability to generate real-time text, code, and explanations across a broad range of disciplines opened new possibilities for student learning and academic support. Its conversational nature allows students to engage with AI in an interactive, iterative process, refining their understanding through dialogue rather than passive content consumption. As a result, researchers have begun investigating how ChatGPT adapts to individual learners’ needs and pace, as well as its broader impact on higher education^12,13^. Nevertheless, concerns about AI systems’ accuracy and students’ over-reliance on them remain central to ongoing debates about AI’s role in education.

One significant area of AI-focused research has been Conversational Tutoring Systems (CTSs), which provide dialog-based learning experiences tailored to individual students. CTSs simulate the role of human tutors, engaging students in structured conversations to clarify concepts, answer questions, and guide problem-solving. Previous studies have demonstrated that dialog-based CTSs improve engagement and motivation in students, particularly in STEM education and language learning^14^. AI-powered tutoring systems have also been studied as a tool for supporting student writing, where they provide feedback on structure, coherence, and argumentation quality^15–17^. Recent findings suggest that key factors such as learner autonomy and interactivity are crucial in fostering student satisfaction with these AI tutors^5^.

A growing body of work is beginning to examine how undergraduate and graduate students use AI-powered tutors and feedback systems^18,4^. These studies suggest that AI’s role in higher education extends beyond structured tutoring to include more flexible, self-directed applications. For example, university students may use AI to brainstorm ideas, refine their arguments, or verify facts, rather than simply answering predefined questions. AI systems such as ChatGPT can also support peer collaboration by helping students generate discussion points, critique each other’s work, and refine their reasoning through AI-assisted argumentation.

Beyond tutoring, AI is increasingly used to provide feedback on student writing. Studies have found that AI-generated feedback, whether used alongside peer critique^6^ or as an alternative to human-generated feedback^8^, can be effective in improving essay-writing quality. AI feedback tools such as ChatGPT offer immediate, constructive comments, allowing students to make real-time revisions to their work^19^. Unlike traditional writing feedback, which often focuses on surface-level corrections, AI-based systems can provide explanations, suggest alternative phrasings, and highlight logical inconsistencies. This immediacy has been particularly useful for students in large classes, where individualized feedback from instructors may be limited. However, while many studies have focused on structured AI feedback systems designed for specific courses, fewer have examined how students employ general-use AI tools such as ChatGPT in unstructured coursework^12^. Other research has examined broader student attitudes toward ChatGPT, exploring how students adopt the tool in different academic contexts^20^, their perceptions of its ethical implications^21^, and the benefits and barriers associated with AI in education^22^.

Despite growing research on AI in education, many studies focus on institutional concerns or controlled experimental settings rather than real-world student behavior in open-ended coursework. Our study addresses this gap by providing a qualitative analysis of student AI use in a large General Education course where AI integration was explicitly permitted rather than restricted. By analyzing students’ documented reflections, we capture the diverse ways they employed AI—from idea generation and revision assistance to content expansion and structural refinement. Additionally, our findings shed light on students’ decision-making processes, including when they chose to rely on AI and when they opted for independent work. These insights offer a valuable snapshot of early AI adoption in higher education and provide guidance for educators seeking to balance efficiency, autonomy, and critical engagement in AI-integrated learning environments^12,18^.

## Theoretical framework

This study examines student AI use through the lens of sociocultural learning theory, particularly Vygotsky’s^23^ concept of the zone of proximal development and tool mediation. In this framework, AI can function as a ‘more expert other’ that supports students in extending their existing abilities and reaching higher levels of proficiency. This theoretical perspective helps us understand how students use AI not just as a technical tool, but as a mediating agent in their learning process. The framework also draws on computer-supported collaborative learning theories^16^, which help explain how students engage with AI as a learning partner rather than merely a text generation tool.

### Methods

The data analyzed in this article were collected from a large (277 student) undergraduate course on sustainability and technology, taught in Spring 2023 at a large research university in the United States. The course satisfied a General Education (GE) requirement in the Science and Technology category, and therefore attracted students from all years (first year through senior) and from 46 different majors across campus. While we know the disciplinary diversity of enrolled students, we do not have access to other demographic information about the participants, which limits our ability to analyze how factors such as age, gender, or educational background may have influenced AI usage patterns. This study was reviewed and approved by the University of California, Irvine’s Institutional Review Board (IRB #6704). The need for informed consent was waived due to its classification as exempt “research conducted in an educational setting involving normal educational practice”^24^. The data used were deidentified and analyzed after the course was completed and therefore were “not likely to adversely impact students’ opportunity to learn or assessment of educators”^24^. All methods were carried out in accordance with relevant guidelines and regulations.

**Fig. 1:**
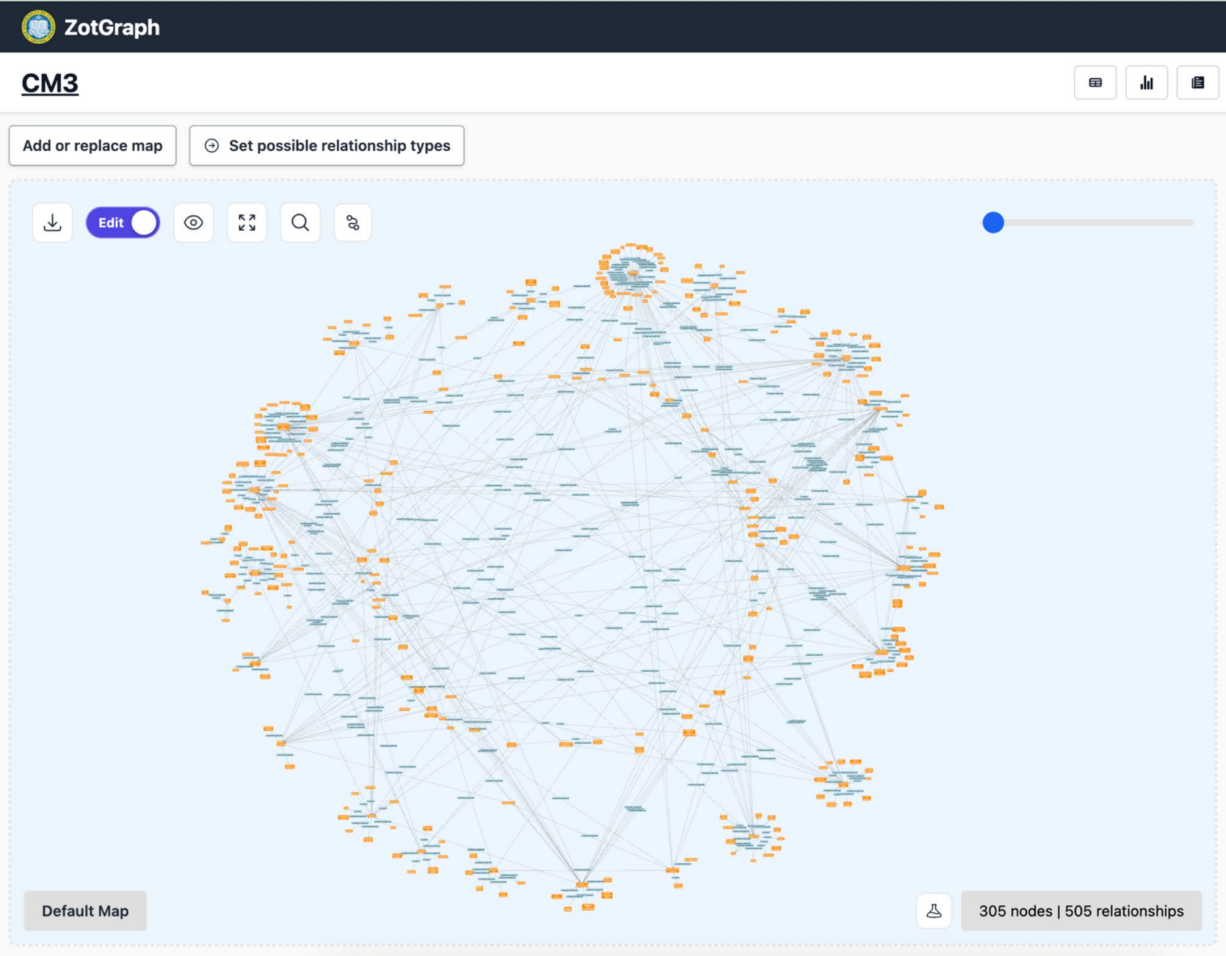
The conceptual network that students worked with in their final project. This network was created via custom software created by our team (http://www.zotgraph.com, version 1.0).

The data for this study come from students’ use of AI in their final course project assignment. This assignment was created by the second author, who was the instructor for the course. This final project built on work that students had completed over the duration of the course. Specifically, students used bespoke software to craft conceptual networks connecting core concepts of the course (e.g., sea level rise, ocean acidification, climate change) with other concepts drawn from different sources (e.g., course materials, students’ majors or daily lives, current events, etc.). For example, a biology student might connect the concept of climate change with the loss of biodiversity. Once students completed their conceptual networks, the teaching staff used the software to merge individual student networks into a larger, collaboratively-generated network featuring 305 concepts connected by 505 relationships (see Fig. 1).

This larger network only included relationships that had been added by at least two students. Merging all concepts and relationships added by only one student would have been prohibitively complex, and substantially reduced the accuracy of content in the shared network. Even with the requirement that a relationship be added by at least two students, there were still various errors on the map. For example, at least two students added that e-waste recycling is positively coupled with e-waste, when in fact the opposite is true (e-waste recycling reduces e-waste).

For their final projects, students were asked to identify various patterns in the larger network, such as causal chains and feedback loops. For each of these patterns, they were asked to include a several-paragraph written description responding to a set of predetermined questions. For example, students were asked to find a feedback loop in the network, describe that feedback loop in prose, and then respond to the following prompt: “Does this feedback loop exist in the real world? If it exists in the real world, please provide evidence of it being discussed in reputable publications (e.g., one or more reputable publications that describe some or all of the linkages in this feedback loop). If it does not exist in the real world, write a paragraph describing how important you think this feedback is. Do you think it has a major impact on climate change or other topics? Or is it trivial, because (for example) the strength of the connections is small, or the topics are unimportant?” (The teaching staff had previously identified “reputable publications” as being either peer-reviewed or from well-reputed news sources.) There were three other portions of the assignment: identifying incorrect relationships, identifying a causal chain, and finding an example of both direct and indirect causality.

Students were permitted to use AI to support their work on these documents. The prompt notified students that they were “welcome (but not required) to use AI to support [their] writing.” It also required students who used AI to “include a section labeled ‘Use of AI’ that describes what system you used, how you used it, what prompts you used, and how you think using AI may have impacted your work on this assignment.” Students were not constrained regarding which AI systems they could use.

Students submitted their final projects and AI documentation with their names attached - there was no anonymous submission option. At the time of data collection in May/June 2023, the teaching staff’s primary goal was pedagogical: to understand how this newly emerged technology (ChatGPT had been released just months earlier) was affecting students’ coursework. The research value of these submissions only became apparent after the course concluded. While students were given permission to use AI and were asked to document that use, the connection between their identifiable information and their AI documentation may have influenced how they reported their AI usage.

Of the 277 students, 39 included explicit written content about their use of AI. Since AI writing detectors are notoriously unreliable, we did not attempt to identify students who used AI but did not document that usage; nevertheless, it is certainly possible that additional students used AI. However, various students’ writing was sufficiently flawed that we are confident that not all students used AI as a grammar revision tool.

The 39 students who described their use of AI did so in a variety of ways. Most students (20) included an explicit section labeled “Use of AI” as requested (with the others labeling it “AI Policy”, etc.), but how they responded to the questions varied widely, from a 9-word response to a 366-word essay. One other student included short blurbs under each of their responses about how they used AI in that particular section. The “Results” section below presents an analysis of the students’ content.

The authors used an iterative process of qualitative coding to analyze the data, using the Dedoose software system (https://www.dedoose.com). In the first round of coding, the second author used a combination of a priori or deductive codes based on elements of composition (e.g., brainstorming, drafting, revising, editing) and inductive codes based on students’ descriptions of how they used AI to code the data using the software Dedoose. The first author provided a definition for each code as well as inclusion and exclusion criteria. For example, the code drafting was defined as “using AI to generate initial text” with inclusion criteria being “any references to *writing a draft*,* composing*,* putting ideas into words*” and exclusion criteria being “any references to improving communication or writing.” Next, the second author independently coded the data using the codes generated by the first author and adding additional codes as needed. Next, the authors looked at the coded data together, attending to data excerpts that were coded differently, and attempting to reach agreement on the codes for those excerpts. If the authors were unable to reach agreement on the code for an excerpt, it was excluded from the data.

### Results

Our final analysis yielded 38 separate codes (samples included in Table 1). We categorized those codes into 6 broader thematic categories: higher order writing tasks, lower order writing tasks, other learning activities, independence, efficiency, and skepticism. In the results section, we present quotes to illustrate specific usages of AI; however, many of these excerpts were simultaneously coded^25^, as illustrated in the table below.

**Table 1:** Sample coding of student responses.

Excerpt	Code(s)	Category
*At various points within this assignment I used ChatGPT to help me formulate more specific ideas and examples*,* correct grammar*,* and choose better language.*	Generating ideas Finding evidence or examples Revising	Higher Order Higher Order Lower Order
*Prompt used: “Is the grammar correct?” Not*,* “Fix the grammar.” I noticed when I ask the AI to fix the grammar it uses new words that are not originally my words*,* but when I ask if it is correct it gives me slight revisions of my words.*	Prompt Revising Independent Writing	Lower Order Independence
*IDEAS*,* CONCEPTS*,* AND SOURCES remain original. AI used to paraphrase my ideas in an academic fashion. AI generated sentences mixed along with original sentences.*	Independent Ideas Paraphrasing Academic Writing Mixture	Independence Lower Order Higher Order
*I used this assignment to double-check if the relationships or casual chains were correct. For example*,* one prompt I gave was “ Is education positively coupled with industrialization?”. If the AI indicated that it was correct*,* then I would look up credible sources related to the relationships to ensure that this relationship is accurate.*	Other Prompt Checking Ideas Skeptical Checking AI	Other Skeptical

#### Higher order writing tasks

Higher order writing tasks require deep understanding of the content, critical thinking, and the ability to synthesize and analyze information. Our data indicate that students used AI software to assist them with some higher order writing and learning tasks. For example, “Understanding Complex Topics” was a prevalent code (24 instances) where students described how they used AI to help them better understand concepts and the relationships between them.

*Using AI had an impact on my work by enabling me to access a vast amount of knowledge and information stored in the model. It allowed me to provide a comprehensive summary of the causal chain and draw upon various concepts and research findings related to the topic.*

*Although I had a decent grasp on how the concepts within the relationships I found were related*,* I prompted ChatGPT to describe the connections in more detail in order to gain a better understanding of the relations.”*

*I initially used ChatGPT AI to summarize possible conceptual connections that I could write about and it helped me see the basic relationships between them.*

*For the direct and indirect causality*,* I asked Chat GPT how all three topics are linked to each other and then proceeded to write my own interpretation.*

Students also reported using AI to help them with “Finding Evidence or Examples” (11 instances).

*Within the feedback loop section of the assignment*,* I asked ChatCPT*(Students used multiple different spellings and capitalizations for ChatGPT, including “ChatCPT” (as in this quote), “ChatGBT”, “ChaptBGT”, “Chatgpt”, and “chatgpt”. We leave the version that students used in each quote.) *to give an example of industrialization leading to capitalism*,* again*,* verifying the example with my own research.*

*Find me real life examples for this causal chain*.

Most instances of “Understanding Complex Topics,” and “Finding Evidence or Examples” related to students using AI to enhance and synthesize their existing understandings of conceptual connections. Students then used these enhanced understandings to write their own interpretations of the material. This points to the valuable role that AI played in facilitating analytical and research-oriented tasks, central to higher order writing.

Some students, however, reported using the AI to help them find “starting point[s] on how concepts were related” and said that the AI gave them “thought provoking answers to the questions, ones that I don’t think that I would come up with myself.” Such examples signal a strong reliance on AI for students’ foundational understandings of the course content.

#### Lower order writing tasks

Lower order writing tasks focus on the mechanics of writing, such as grammar, punctuation, and spelling, which are essential for polishing a piece of writing. Our analysis found that lower order writing tasks were mentioned much more frequently than higher order tasks. The most frequent codes within this category were “Revising and Editing” (47 instances). While revising and editing are rhetorically distinct, for the purposes of this analysis, it was necessary to collapse these categories because it was not always possible to tell if a student was actually using AI for revision or simply using the word “revise” to describe editing. Students reported using AI for “revising and editing” to help them better express their existing ideas. For example:

*I entirely wrote it out but had AI re-write my chicken scratch and feel that it significantly enhanced the quality of my work.*

*I think the only impact AI had on my work in this assignment was just making my responses precise and written at a higher level in terms of vocabulary and flow.*

*It would then take a few seconds and generate a paragraph that didn’t change any of the content I had written but rather just made adjustments to make my responses more academic-sounding.*

*Then in ChatGPT I put “strengthen this writing” followed by my paragraphs placed in parentheses.*

*The original information served as the foundation for the project*,* with AI offering modifications to enhance clarity*,* coherence*,* and precision.*

*To correct grammar and readability*.

*AI used to paraphrase my ideas in an academic fashion.*

Subcodes helped us to identify specific aspects of “Revising and Editing” that students were using AI to support. Students reported using AI to help them with their word choices and to help them make their writing sound more academic, illustrating a significant use of AI in refining and editing academic texts. These findings are indicative of the pivotal role AI plays in enhancing the technical quality of students’ written output.

#### Ghostwriting

Some students reported using AI to “ghost write” their texts (24 instances); however, the degree to which they relied on the AI to compose for them varied significantly. For example, some students seemed to rely heavily on the AI to write text for them.

*The system that I used is ChatGPT. I have always heard of this system but never used it before*,* I must admit I was very surprised by the advancement of this tool. ChatGPT gives you drafts or literal paragraph responses of whatever you type in the chat box*,* it makes connections easier to assemble.*

*I provided the system with the initial prompts and guidance to structure the information.*

*…it really helped me add some analysis to things that I did not know how to write about.*

*I fed AI a couple different quotes and asked it to integrate it with my answer.*

Other students reported using the AI to help them get past writer’s block.

*When experiencing writer’s block or struggling with a specific word*,* I found the “complete my sentence” command to be helpful.*

*Chat GPT allowed me to break out of writer’s block by being able to bounce ideas off of it.*

*Sometimes I felt stuck because I am a senior in my last quarter but it just refreshed my ideas and helped me write.*

Other specific tasks included expanding their text to meet the word count and synthesizing their ideas into concluding paragraphs.

#### Beyond writing: efficiency and independent learning

Interestingly, our data reveal uses of AI that transcend traditional writing tasks. “Efficiency” (14 instances) emerged as a notable theme, with students using AI to save time and streamline the academic work process. Additionally, the emphasis on “Independent Ideas” (12 instances) and “Independent Writing” (8 instances) suggest that AI tools are perceived as facilitators of creativity and independent thought, rather than mere aides for rote tasks. As an example, one student wrote, “I want to clarify that this assignment is based solely on my own ideas and produced independently.” Other students wanted to clarify that, even though they used the software for editing and proofreading, they wanted to ensure that they were able to retain their own words.

*Prompt used: “Is the grammar correct?” Not*,* “Fix the grammar.” I noticed when I ask the AI to fix the grammar it uses new words that are not originally my words*,* but when I ask if it is correct it gives me slight revisions of my words.*

In addition, students reported using AI to conduct their research. Specifically, they used AI to check their ideas, help define terms, find more information about specific concepts, search for evidence or examples to support their independent ideas and arguments, and to find relevant references.

#### Student perspectives on AI

The occurrence of “Skeptical” (12 instances) as a code reveals a critical perspective towards the use of AI among some students. Students expressed concerns about the appropriateness of the AI’s writing and the outdated nature of AI data.

*I didn’t just completely use what the AI wrote however because every time I would run into a problem where the generated response often sounded repetitive so I picked and chose portions of the generated response to incorporate into what I had already written.*

*I did not ask it to produce any specific examples of the relationships in the real world because the bot’s information is only as recent as 2021 and has often been found to make up random “facts.”*

As can be seen in the previous quote, students were also skeptical of the accuracy and reliability of the material produced by the AI. In general, they expressed concerns about ChatGPT making up facts and inventing references.

*For the rest of the assignment I tried to use it also*,* but most of the citations given were wrong or did not exist*.

*However*,* it’s important to note that while the AI model is a powerful tool*,* it may not always capture the nuances or context-specific details accurately. It is still crucial to exercise critical thinking and verify information from primary sources when necessary.*

*it still cannot replace human for now. It’s not accurate enough and to me just not trust worthy.*

To address this, students described “verifying the example with [their] own research” and using a mixture of their own sentences and those of ChatGPT.

## Discussion

Higher order writing tasks can support students’ abilities to engage critically with subject matter, requiring not just a grasp of existing knowledge but an ability to evaluate, synthesize, and generate new insights. These tasks are foundational in fostering critical thinking and analytical skills, which are pivotal in academic and professional settings. In our study, the data indicate a significant dependence on AI software among students, especially in the realms of “Finding Evidence or Examples,” “Finding Information,” and “Understanding Complex Topics.” This reliance on AI for sourcing and digesting complex material aligns with recent findings by Wambsganss et al.^5^, who found that university students particularly value AI tools that support autonomous learning and complex task completion. However, while Wambsganss et al. focused on a structured writing tutor system, our findings suggest students apply similar strategies even in open-ended contexts.

Our findings both complement and extend previous research on AI-assisted learning in higher education. For example, Banihashem et al.^6^ found that students effectively used AI-generated feedback to improve their essays, but primarily focused on technical aspects of writing. In contrast, our study reveals that when given freedom to use AI as they choose, many students leverage it for deeper conceptual understanding and evidence gathering. This difference suggests that structured AI systems might be unnecessarily constraining student engagement with AI tools. Similarly, while Escalante et al.^8^ documented student preference for AI feedback in specific writing tasks, our findings show students actively discriminating between tasks they consider appropriate for AI assistance versus those requiring independent thought.

The utilization of AI for “Independent Research” suggests a proactive approach by students, using AI to dig deeper into subjects and find relevant scholarly articles, datasets, and other resources that are beyond the superficial layers of information accessible through conventional search engines. This aspect of AI use may highlight a shift towards more autonomous learning, where students harness technology to drive their inquiry and extend their research boundaries.

Similarly, the frequency with which students reported using AI for “Finding Information” reflects the pivotal role of AI in sifting through information, verifying facts, and identifying the most relevant data. This process is crucial in building a solid foundation for any higher order writing task, where substantiated arguments and evidence-based claims form the backbone of quality academic writing.

Furthermore, the recurrent use of AI for “Finding Evidence or Examples” and “Understanding Complex Topics” underscores its significance in enhancing students’ research capabilities. “Finding Evidence or Examples” is vital for supporting arguments, illustrating points, and grounding discussions in concrete instances or findings, thereby enriching the academic discourse. “Understanding Complex Topics”, on the other hand, involves breaking down content into comprehensible segments, a process where AI can provide explanations, simplifications, or different perspectives that aid in grasping challenging concepts.

Collectively, these findings demonstrate the integral role that AI can play in facilitating higher order writing tasks by enabling access to, and interpretation of, complex information. Students are not merely passive recipients of AI-generated content; instead, they are engaging actively with AI-based tools to augment their research processes, enhance comprehension, and construct well-informed, analytically-robust academic texts. This blending of student effort and the capabilities of AI for tackling higher order writing tasks is emblematic of a shifting educational paradigm, where AI can act as a “more expert other” that allows students to work within the zone of proximal development^23^. Specifically, students use the AI to help them build on what they already know, ideally engaging in critical thinking and deep learning in the process, as they evaluate the AI-generated content against what they have learned in class and decide how best to integrate such content with their pre-existing knowledge in ways that meet the requirements of their assignments.

Lower order writing tasks, though often perceived as merely the final polishing steps in the writing process, are crucial for conveying ideas clearly and professionally. These tasks encompass the mechanics of writing, including grammar, punctuation, and spelling, which are fundamental for enhancing the readability and credibility of academic writing. Our analysis sheds light on the potential that AI offers for supporting students in these essential components of the writing process, with “Revising and Editing” standing out as the most prevalent code in this category. This finding aligns with Gombert et al.‘s^19^ research showing students’ strong preference for AI assistance with technical writing aspects. However, while Gombert et al.‘s study focused on structured essay assessment, our findings reveal students independently choosing to use AI primarily for these lower-order tasks even when given complete freedom in their AI use.

The prevalence of AI use for editing and revision in our study both confirms and extends previous research. For example, Banihashem et al.^6^ found that students valued AI-generated feedback for technical improvements, similar to our findings. However, our results suggest a more nuanced approach—students actively distinguished between using AI for mechanical improvements versus maintaining their original voice and ideas. This selective approach to AI assistance wasn’t observed in previous studies of structured AI writing systems, suggesting that when given agency, students develop more sophisticated strategies for integrating AI into their writing process.

The act of “Revising” involves more than just making minor edits; it requires students to review the text carefully to improve its flow, clarity, and overall structure. Students’ use of AI for revision suggests a strategic approach to refining their arguments, reorganizing information for better coherence, and ensuring that their writing meets the expected academic standards. AI tools can offer suggestions for restructuring sentences, enhancing word choice, and identifying logical inconsistencies, thereby providing support for students aiming to improve their compositions.

“Proofreading,” on the other hand, focuses on correcting surface errors in writing, such as typos, misspellings, and grammatical mistakes. The frequent use of AI for proofreading points to its effectiveness in catching errors that might elude the human eye, ensuring that documents are polished and error-free. This is an important task, as even minor mistakes can distract readers and detract from the writer’s credibility. By automating the laborious process of proofreading, AI allows students to focus more on the content and substance of their work.

These findings illustrate the potential role of AI in students’ writing process. AI does not replace human judgment, but rather complements students’ efforts in crafting well-written, technically sound documents. By using AI for lower order writing tasks, students are able to produce texts that are not only conceptually strong but also flawless in their execution. This dual focus on content and form is essential for achieving excellence in academic writing.

We also note that, for students to benefit the most from using AI for revising and proofreading, they should have guidelines for the sort of prompts to use. Specifically, these should be prompts that generate feedback on the grammaticality of students’ writing rather than rewriting students’ work. Our analysis demonstrated that some students used these types of prompts, such as the student who shifted their prompt to elicit suggestions for slight revisions of their words. This sort of feedback provides students with an opportunity to see grammatical trouble sources and actively engage in correcting them.

Our analyses revealed complex patterns in how students interact with AI technologies in their academic endeavors. The concept of “Efficiency” emerged prominently in our codes, aligning with Strzelecki’s^20^ findings on student AI adoption patterns. However, while Strzelecki found general acceptance of AI tools, our study revealed a more nuanced approach where students strategically used AI to streamline specific tasks while maintaining their intellectual independence. This selective efficiency mirrors Thi Thuy’s^22^ findings about students’ careful navigation of AI benefits and limitations, though our results suggest even more sophisticated decision-making about when and how to leverage AI support.

The students in our study went beyond simple time-saving uses, demonstrating what Wambsganss et al.^5^ described as “adaptive engagement” with AI tools. By using AI to handle mechanical tasks like proofreading and quote integration, students reported being able to focus more on critical thinking and complex analysis. This finding extends previous research by showing how some students, when given agency, naturally develop workflows that preserve their intellectual engagement while leveraging AI for efficiency. Unlike studies of structured AI systems^6,8^, our results suggest that students can effectively self-regulate their AI use to maintain both productivity and learning quality.

Moreover, the data reveal a substantial emphasis on student independence, indicating a perception of AI not merely as a tool for task completion but as an enabler for students’ own creative and independent thinking and learning. The use of AI to help students generate “Independent Ideas” points to its capacity to provoke thought, offer unique perspectives, or suggest novel solutions that might not have been immediately apparent to the student. This can be particularly valuable in brainstorming sessions, conceptual development phases, or when seeking to explore alternative approaches to problem-solving. Similarly, AI can assist in overcoming writer’s block by suggesting sentence starters or offering different ways to phrase ideas, thereby aiding in the translation of complex thoughts into coherent written form. This aspect of AI use has the potential to foster a sense of autonomy in students by offering them an ever-present “sounding board” and “peer” reviewer for their ideas.

Despite the many ways that students relied on AI for their projects, our data indicate a sophisticated skepticism about these systems. This finding both aligns with and extends previous research on student AI perceptions. While Farhi et al.^21^ documented general student concerns about AI ethics and reliability, our study reveals more specific and nuanced forms of skepticism. Students in our study demonstrated what Strzelecki^20^ calls “critical technology acceptance,” actively evaluating AI outputs against their course knowledge and making informed decisions about what to include or exclude. This approach extends beyond the basic digital literacy described by Lamb et al.^26^ to encompass sophisticated evaluation of AI-generated content.

Particularly noteworthy was students’ careful maintenance of their intellectual independence, a finding that adds new dimensions to existing research on AI adoption in higher education. Unlike studies showing either broad acceptance^22^ or resistance^2^ to AI tools, our results suggest students can develop balanced approaches that leverage AI’s benefits while preserving their academic autonomy. This selective engagement may reflect the explicit permission given for AI use in our study, contrasting with contexts where AI use is either mandated or restricted.

The concerns expressed by students indicate that educators and academic institutions need to better address the role of AI technology in educational settings. Teachers should engage students in conversations about AI, providing resources that enable them to critically evaluate these technologies and their applications. By exploring the capabilities and limitations of AI systems, students can learn to make well-informed decisions about how to effectively and ethically incorporate them into their academic work. In addressing students’ concerns head-on, the academic community can work towards integrating AI in a manner that both enhances the learning experience and maintains academic integrity.

While our study suggests that AI may provide effective support for students’ learning in higher education contexts, there are some limitations to consider. First, our data consists of students’ self-reported use of AI in submissions that were not anonymous. Students were asked to document their AI usage as part of their identified final project submissions, which may have influenced how they characterized their interactions with these tools. Social desirability bias could have led students to underreport their use of AI or to emphasize certain types of AI usage (like proofreading and editing) that they perceived as more academically acceptable while downplaying other uses. The lack of anonymity may have amplified this effect, as students might have been more cautious about documenting AI use when their names were attached to their submissions. Some students may have opted not to report their AI use at all, fearing potential academic consequences despite explicit permission to use AI for the assignment. Future research might benefit from alternative methods of documenting AI use, such as automated tracking of AI interactions, anonymous reporting mechanisms, or structured interviews that explicitly address students’ comfort in discussing their AI use.

Second, our study captures a specific moment in time - early 2023, just months after ChatGPT’s release. Student approaches to AI use likely evolved as they gained more familiarity with these tools and as the tools themselves improved. The rapid pace of AI development means that patterns we observed may not reflect current student practices.

Third, our findings come from a single course at one university, focused on sustainability and technology. The nature of the final project - analyzing conceptual networks - may have led to different AI usage patterns than might emerge in other types of courses or assignments. Students in this General Education course also came from diverse academic backgrounds, which could have influenced their comfort with and approaches to using AI tools.

Fourth, our sample size of 39 students who documented their AI use represents only a portion of the course enrollment. While we know these students came from various majors, we lack demographic information that could help us understand how factors such as age, year in school, or prior technology experience might influence AI usage patterns.

Finally, this study’s original purpose was pedagogical rather than research-oriented. Had we designed it primarily as a research study, we might have implemented different data collection methods, such as structured interviews or surveys, to gather more detailed information about students’ decision-making processes regarding AI use.

Future research might address these limitations through larger-scale studies across multiple institutions and course types, longitudinal analyses of how AI use evolves over time, and mixed-methods approaches that combine self-reporting with other data collection strategies.

## Conclusion

AI is highly likely to play a role in the future of education; it is critical that scholars and practitioners develop an understanding of how to enable AI to benefit student learning rather than just giving students a shortcut to generating content without engaging in the learning process. Our qualitative analysis of undergraduate students’ use of AI in a final course project revealed that AI is transforming the educational landscape in a variety of ways. Students harnessed AI for many different purposes, ranging from lower order writing tasks like proofreading and editing to higher order tasks such as understanding complex topics, finding evidence and examples, and conducting independent research. AI also served to enhance efficiency and support students’ independent thinking and learning. While AI tools are largely embraced for their efficiency and support in generating ideas and understanding complex topics, a layer of skepticism among users points to a need for ongoing dialogue about the ethical use and potential limitations of these technologies in educational settings. Our study contributes to an understanding of students’ use of AI in a specific academic context; nevertheless, further research is needed to understand the implications of AI use in education across longer time periods, and to develop best practices for its effective and ethical implementation.

## Data Availability

The dataset on which this study is based is available here: https://osf.io/6c5xe/.
